# Existence and Quality of Data on Control Programs for EU Non-regulated Cattle Diseases: Consequences for Estimation and Comparison of the Probability of Freedom From Infection

**DOI:** 10.3389/fvets.2021.689375

**Published:** 2021-07-19

**Authors:** Egle Rapaliute, Annika van Roon, Gerdien van Schaik, Inge Santman-Berends, Xhelil Koleci, Madalina Mincu, Jörn Gethmann, Beate Conrady, Tanja Knific, Jaka Jakob Hodnik, John Berezowski, Luís Pedro Carmo, Aurélien Madouasse, Attila Tarpai, Anton Gerilovych, Alvydas Malakauskas, Blagica Sekovska, Christine Fourichon, Emmanouil Kalaitzakis, Franz-Ferdinand Roch, Hans Houe, Katarzyna Dudek, Kerli Mõtus, László Ózsvári, Lina Costa, Maria Guelbenzu-Gonzalo, Madeleine K. Henry, Mentor Alishani, Nicola Pozzato, Petter Hopp, Ramon Juste, Sam Strain, Rene Mandelik, Štefan Vilček, Tiina Autio, Lena-Mari Tamminen, Céline Faverjon

**Affiliations:** ^1^Department of Veterinary Pathobiology, Faculty of Veterinary Medicine, Veterinary Academy, Lithuanian University of Health Sciences, Kaunas, Lithuania; ^2^Department of Population Health Sciences, Unit Farm Animal Health, Faculty of Veterinary Medicine, Utrecht University, Utrecht, Netherlands; ^3^Department of Epidemiology, Royal GD, Deventer, Netherlands; ^4^Faculty of Veterinary Medicine, Agricultural University of Tirana, Tirana, Albania; ^5^Research and Development Institute for Bovine Balotesti, Ploiesti, Romania; ^6^Institute of Epidemiology, Friedrich-Loeffler-Institut, Greifswald, Germany; ^7^Unit of Veterinary Public Health and Epidemiology, Institute of Food Safety, Food Technology and Veterinary Public Health, University of Veterinary Medicine Vienna, Vienna, Austria; ^8^Complexity Science Hub Vienna, Vienna, Austria; ^9^Department of Veterinary and Animal Sciences, University of Copenhagen, Frederiksberg, Denmark; ^10^Veterinary Faculty, University of Ljubljana, Ljubljana, Slovenia; ^11^Veterinary Public Health Institute, Vetsuisse Faculty, University of Bern, Bern, Switzerland; ^12^INRAE, Oniris, BIOEPAR, Nantes, France; ^13^Section of Epidemiology, Norwegian Veterinary Institute, Oslo, Norway; ^14^Institute for Experimental and Clinical Veterinary Medicine, Kharkiv, Ukraine; ^15^Faculty of Veterinary Medicine, Ss. Cyril and Methodius University, Skopje, North Macedonia; ^16^Clinic of Farm Animals, School of Veterinary Medicine, Aristotle University Thessaloniki, Thessaloniki, Greece; ^17^Department of Cattle and Sheep Diseases, National Veterinary Research Institute, Pulawy, Poland; ^18^Institute of Veterinary Medicine and Animal Sciences, Estonian University of Life Sciences, Tartu, Estonia; ^19^Department of Veterinary Forensics and Economics, University of Veterinary Medicine Budapest, Budapest, Hungary; ^20^Polytechnic Institute of Portalegre, Praça Do Município 11, Portalegre, Portugal; ^21^Animal Health Ireland, Carrick-on-Shannon, Ireland; ^22^Epidemiology Research Unit, Department of Veterinary and Animal Science, Northern Faculty, Scotland's Rural College, Inverness, United Kingdom; ^23^Veterinary Department of the Faculty of Agriculture and Veterinary, University of Prishtina, Prishtina, Kosovo; ^24^Istituto Zooprofilattico Sperimentale delle Venezie, Legnaro, Italy; ^25^Department of Animal Health, NEIKER-Basque Institute for Agricultural Research and Development, Arkaute, Spain; ^26^Animal Health and Welfare Northern Ireland, Dungannon, United Kingdom; ^27^Department of Epizootiology, Parasitology and Protection of One Health, University of Veterinary Medicine and Pharmacy, Kosice, Slovakia; ^28^Veterinary Bacteriology and Pathology Unit, Finnish Food Authority, Kuopio, Finland; ^29^Department of Clinical Sciences, Swedish University of Agricultural Sciences, Uppsala, Sweden; ^30^Ausvet Europe, Lyon, France

**Keywords:** animal health data, cattle, control programs, non-regulated diseases, output-based, proof of freedom

## Abstract

Some European countries have successfully implemented country-specific control programs (CPs) for infectious cattle diseases that are not regulated or are regulated only to a limited extent at the European Union (EU) level. Examples of such diseases include bovine viral diarrhea (BVD), infectious bovine rhinotracheitis (IBR), and Johne's disease (JD). The CPs vary between countries in the design and quality of collected data as well as methods used to detect infection and estimate prevalence or probability of freedom from infection. Differences in disease status between countries and non-standardized approaches to assess freedom from infection pose a risk for countries with CPs for non-regulated diseases as infected animals may influence the progress of the disease control or eradication program. The implementation of output-based standards allows estimation and comparison of the probability of freedom for non-regulated cattle diseases in European countries. The aim of the current study was to assess the existence and quality of data that could be used for estimating freedom from infection in European countries. The online data collection tool was sent to 32 countries participating in the SOUND control COST Action and was completed by 24 countries. Data on cattle demographics and data from CPs of IBR and BVD exist in more than 50% of the response countries. However, data describing risk factors and CP of JD was reported as existing in <25% of the countries. The overall quality of data in the sections on demographics and CPs of IBR and BVD were evaluated as “good”, but risk factors and JD data were mostly evaluated as “fair.” Data quality was considered less good mainly due to two quality criteria: accessibility and accuracy. The results of this study show that the quantity and quality of data about cattle populations and CPs are relatively similar in many surveyed countries. The outcome of this work provides an overview of the current situation in the European countries regarding data on EU non-regulated cattle diseases and will further assist in the development and implementation of output-based standards.

## Introduction

Infectious animal diseases are known to be a risk to international trade and public and animal health. To benefit from international trade and provide legitimate protection from animal diseases and zoonoses, countries must comply with the guidelines of the World Trade Organization Agreement on Sanitary and Phytosanitary Measures and the requirements of other standard-setting organizations, such as the World Organization for Animal Health (OIE) and/or the European Union (EU) (1–4). To demonstrate that a region or country is a safe trading partner for animals and animal products, it is necessary to prove freedom from disease (2). In the EU, international standards of surveillance to achieve desired proof of freedom have been developed for some important cattle diseases, e.g., bovine tuberculosis and bovine brucellosis (3). However, for other diseases listed as important for international trade by the OIE (5), such as Johne's disease (JD), infectious bovine rhinotracheitis (IBR), and bovine viral diarrhea (BVD), there are no or limited international standards for proving freedom of disease. Nevertheless, some European countries have successfully implemented country- or region-specific control programs (CPs) for these EU non-regulated diseases. Because of the lack of international standards, the CPs are very diverse, and their outputs are generally difficult to compare, impairing international trade (6–8).

In recent years, output-based standards have been successfully developed and implemented in animal health surveillance (9–15). They appear to be an attractive alternative to input-based standards for EU non-regulated infectious cattle diseases for several reasons. Input-based standards mean that, to be considered free from infection, countries must carry out specified surveillance activities, such as achieving a certain sampling frequency or a minimum sample size or using recommended diagnostic tests (16). On the other hand, output-based standards allow the flexibility to use a wide range of surveillance activities to reach a predefined output (i.e., probability of freedom from infection), supporting the development of cost-effective and efficient surveillance systems (15, 16). Countries with existing CPs for EU non-regulated diseases would then only need to make sure that their surveillance activities are able to achieve a certain level of confidence of freedom (i.e., output) without changing their whole surveillance system to meet prescribed surveillance strategies (i.e., input) (16). The output-based standards more easily adapt to country-specific conditions and better reflect the country-specific disease status (13).

However, the development and implementation of output-based standards to assess the probability of freedom from infection come with challenges. Methods developed for demonstrating freedom from infection using multiple complex data and surveillance activities include scenario tree models and Bayesian models (12, 17, 18). These methods require a large amount of good-quality data to accurately model confidence of freedom from infection (19). The required data have been described before in projects, such as STOC free (Surveillance Tool for Outcome-based Comparison of FREEdom from infection) and RISKSUR (Risk-based animal health surveillance systems), which worked on developing and encouraging output-based standards for animal health (7, 20). Such data include a broad spectrum of information describing the cattle industry, disease introduction risks, biosecurity levels, and existing disease control programs. Only a small portion of these data are routinely collected by the European Commission [e.g., Animal Disease Notification System, Trade Control and Expert System (TRACES), and the OIE World Animal Health Information System] for epidemiological analysis of disease outbreaks, risk analysis, or general statistical information. At the time of writing this paper, the availability of the remaining portion of the data needed to estimate the probability of freedom from infection is unknown. This is especially a concern as good-quality data are likely to be more available for EU regulated diseases compared with EU non-regulated diseases. In addition, even though EU member countries are obliged to collect some of these data on a regular basis, methods of collection and sources of data most likely differ. Recent results from the SIGMA project provide a good overview of the diversity of the animal health data sources network in the EU (21), but the level of heterogeneity in the data collected for different CPs in different countries remains unknown. Understanding the data heterogeneity is the first step toward the use of output-based standards for proving freedom from EU non-regulated infectious diseases.

The COST Action (CA17110) “Standardizing OUtput-based surveillance to control Non-regulated Diseases in the EU” (SOUND control) aims to support output-based disease surveillance initiatives and develop a framework that could be used to estimate the confidence in freedom from EU non-regulated infectious cattle diseases (22, 23). SOUND control covers 32 countries and provides a great opportunity to assess at a large scale the data currently available for estimating the probability of freedom from infection for EU non-regulated cattle diseases and provide recommendations to support the future development of output-based standards.

Our study was conducted within the scope of the SOUND control Working Group 2 (WG2) activities (22) and aimed to (i) provide an overview of the existence of potential data required as inputs for estimating freedom from infection in the 32 Action member countries, (ii) evaluate and compare the quality of these data using a standardized approach, and (iii) review data sources of available data. JD, IBR, and BVD are among the diseases most frequently targeted by CPs implemented in the Action member countries (23). Therefore, we used these three EU non-regulated diseases as case studies.

## Materials and Methods

### Online Data Collection Tool

A thorough description of the online data collection tool, including its development, key lessons learned during the process, and definitions of the variables, can be found in van Roon et al. (24). The online data collection tool was designed using LimeSurvey software (25).

**Figure 1:**
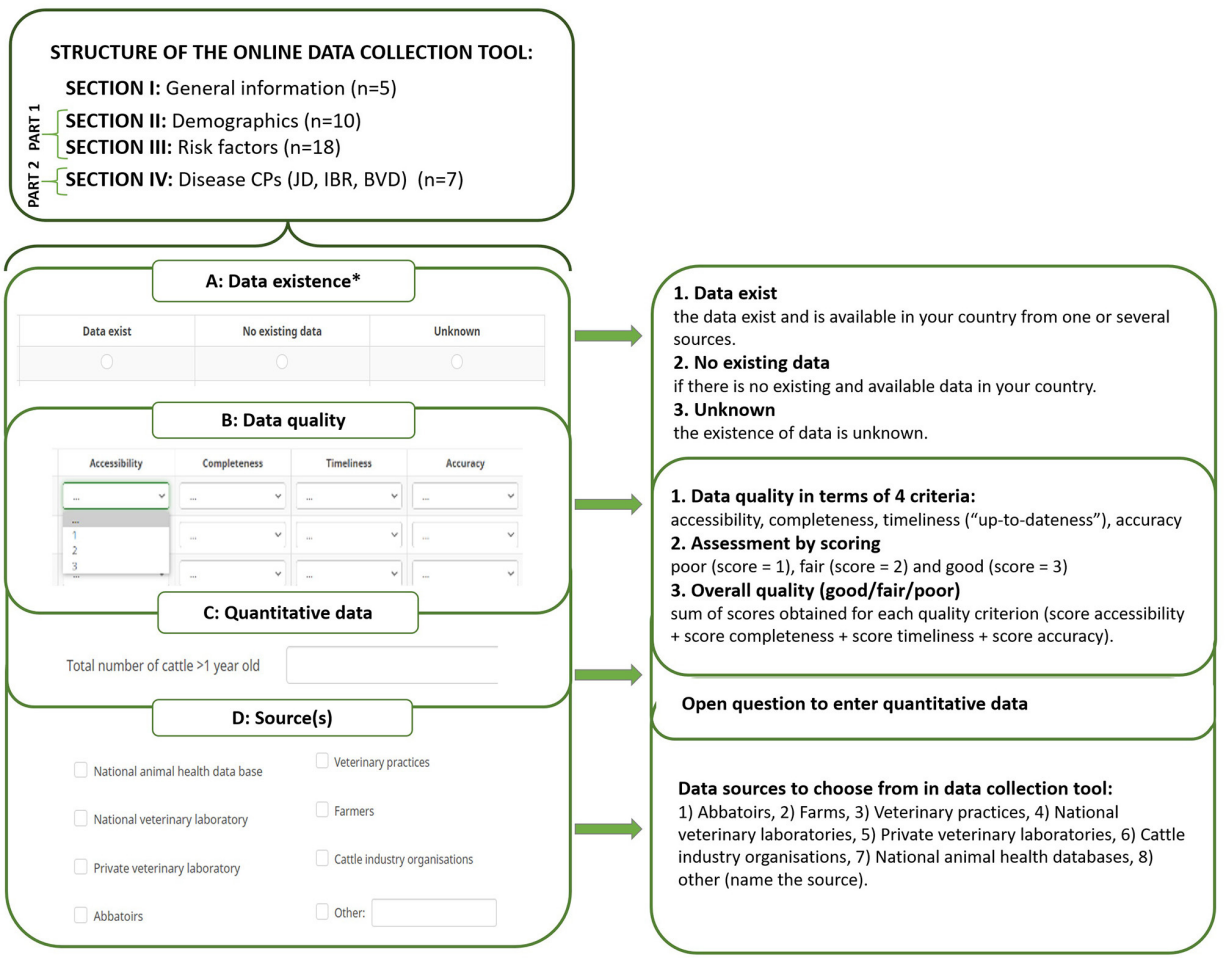
The structure of the online data collection tool that was used to overview data related to control programs of infectious cattle diseases among 32 SOUND control COST Action member countries. ^*^, mandatory part; *n*, number of variables *per section*.

Data from the previous year (or the most recent available) were requested for data collection. Briefly, the online data collection tool was divided into two main parts with four sections and (Figure 1):

- *I. General information*. This section included five basic questions about the time period of the data assessed in the questionnaire, country, contact information of the respondent, and definitions of dairy and beef cattle.
  Part 1:

- *II. Demographics*. This section included 10 questions about the cattle population in the country or region considered in the questionnaire: number of cattle and herds, average herd size, number of births, number of herds with calves, cattle density, and number or percentage of farms with small ruminants and mixed farms (mixed farms are defined as all dairy herds that also have a type of beef cattle, such as veal calves, suckler cattle, etc.).
- *III. Risk factors*. This section included 18 questions about possible risk factors for disease introduction into a cattle herd, such as purchasing, grazing, breeding, housing of calves, control and management of manure, rodent and vector control, transport, disinfection, and equipment on the farm.
  Part 2:

- *IV. Disease control programs and testing strategies for JD, IBR, and BVD*. For each disease, respondents were asked to indicate if a CP for the disease existed in their country. A positive response was followed by seven questions on the number of herds participating in the CP, number of herds tested for the selected disease, animal-, and herd-level prevalence of the selected disease, herds that have a free status for the selected disease, and the number of herds that identified infected animals.

Each question was followed by four additional subquestions related to data existence, data quality, quantitative data, and data sources used to obtain information (Figure 1). The only mandatory question was about the existence of data. Data quality was evaluated using the “Data quality evaluation tool” presented in van Roon et al. (24), which included four criteria (Figure 1B):

- *Accessibility*. The availability of data. This criterion is important because it provides information about how data were collected, for what purpose, and how readily the data can be accessed (e.g., data exist but can be accessed only by combining multiple data sources).
- *Timeliness*. Often described as “up-to-dateness,” it varies depending on the purpose for which the information is required. It is important to evaluate the timeliness to determine whether the information reflects the most recent information.
- *Completeness*. Refers to whether there are missing and/or unknown data fields in the database (e.g., for the variable “number of cattle/herds in territory,” completeness would represent the percentage of farmers entering this information in the data base: 85% of all farmers having filled in the data means completeness of this variable is 85%).
- *Accuracy*. Aims at assessing to what extent the stored values for an object are the correct values (e.g., when data validation procedures are implemented on a regular basis, it is more likely that data is accurate).

Data quality was evaluated using a standardized scoring method (Figure 1B) (24). First, for each quality criteria, a score of three (“good”), two (“fair”), or one (“poor”) was given. Second, the overall quality of the available data was then calculated using the sum of the scores obtained for each quality criterion. Overall quality was also defined as “good” (sum 9–12), “fair” (sum 5–8), or “poor” (sum 1–4).

In addition to data quality, the data source for each question was collected. Respondents were able to choose one or multiple answers from a predefined list of potential data sources, including the option “other,” for which the respondent provided the name of the data source (Figure 1D). Participants were also allowed to submit quantitative data associated with each question (not analyzed in this study) (Figure 1C). Data relating to all cattle (i.e., dairy and beef cattle together) were requested, but it was also possible to submit data separately if needed.

### Data Collection and Management

The SOUND control consortium included representatives by country: one management committee member and one or more management committee substitutes and/or workgroup members. Members who were participating in the workgroups related to data about CPs, one person per country, were responsible for providing the data for their respective country. Almost all participants had a doctoral degree in the field of veterinary sciences or epidemiology and most of them worked in the field of cattle health with a focus on infectious diseases or in surveillance and control of cattle infectious diseases. Depending on their knowledge, the participants could either collect all information to fill in the questionnaire themselves or ask others in their country to help them fill in the questionnaire. Thirty-two SOUND control member representatives were invited by email in July 2020 to fill in the online data collection tool. Three reminders were sent at the end of August, September, and November 2020. The deadline to submit data in the data collection tool was closed in December 2020.

Fully or partially completed questionnaires were extracted from LimeSurvey software to Microsoft Excel 365 and then imported into the R Statistical software, version 3.6.2 (26, 27) for analysis. Descriptive analysis was performed using the “dplyr” package in R (28). Data analysis was structured by country and type of question to calculate and assess (i) the number and proportion of existing data, (ii) the overall quality and quality by criterion of existing data, and (iii) the variety of sources used to obtain data. The R package “ggplot2” was used to visualize the results (29).

## Results

### Response Rate

**Figure 2:**
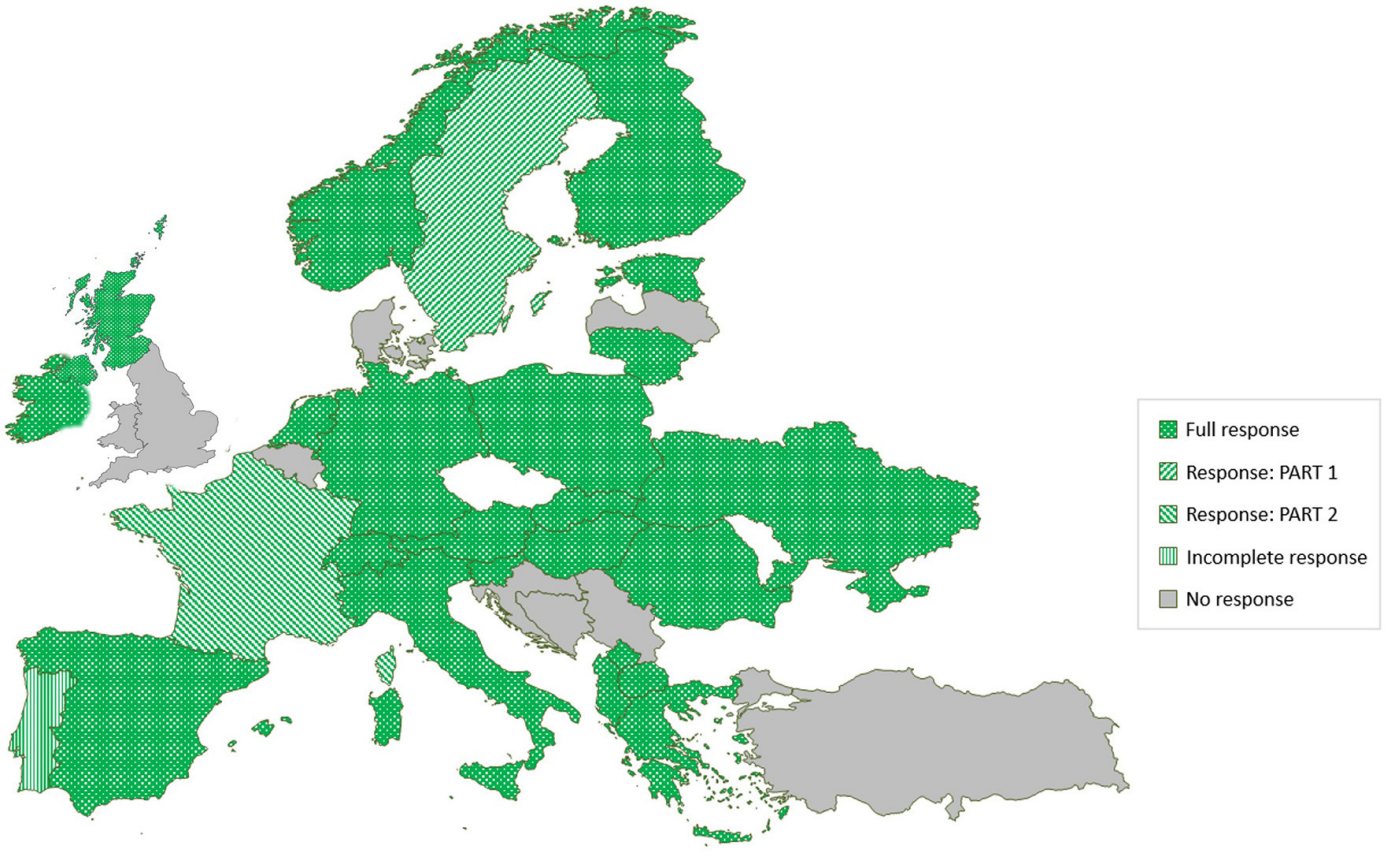
The response to an online questionnaire study on data collection related to EU non-regulated cattle diseases among 32 SOUND control COST Action member countries.

Twenty-four out of 32 of the SOUND control countries completed the online data collection tool (Figure 2). Twenty-two countries fully completed the tool (“Full response”), one country completed only the first part (“Response: PART 1”), and another country completed only the second part (“Response: PART 2”). One country did not fully complete any part of the tool (“Incomplete response”), and seven countries did not respond to the invitation (“No response”). Twenty-two countries reported providing the latest data of 2019–2020, and two countries provided older data (from 2017 to 2018). Regarding existence of CPs, 15 countries reported the existence of a CP and answered questions about IBR, 14 for BVD, and 11 for JD.

### Definitions of Dairy and Beef Cattle

All descriptions of dairy cattle involved characteristics related to milk production and breed, e.g., “deliver milk,” “used in dairy production,” “pure dairy cattle breed,” “farms main income from milk production.” The definitions of beef cattle were less specific. Three countries indicated that there is no definition of beef cattle in their country. When a definition was available, beef cattle were often reported as “veal, beef, and fattening cows,” but sometimes as “all cattle excluding dairy.” Seven countries reported official definitions of dairy and beef. However, the rest of respondents did not specify that the provided definitions were official.

### The Existence of Data

More than 70% of the cattle demographics data investigated in our study were reported as existing in the 24 response countries (Table 1). However, this was only true for 24% of data regarding risk factors (Table 1). Instead, data were reported as “not existing” (38%) or “unknown” (38%).

**Table 1:** Results of the response to questions on existence of data relevant for estimating freedom from infection in cattle in an online questionnaire study among 24 European countries.

**Section**			**Data exist**^**b**^	**No existing data**^**b**^	**Unknown**^**b**^
II. Demographics		(*n =* 24)	75.0% (18)	8.3% (2)	12.5% (3)
III. Risk factors		(*n =* 24)	25.0% (6)	37.5% (9)	37.5% (9)
IV. Disease control programs^a^	JD	(*n =* 11)	36.4% (4)	27.2% (3)	36.4% (4)
	IBR	(*n =* 15)	66.7% (10)	13.3% (2)	20.0% (3)
	BVD	(*n =* 14)	71.5% (10)	21.4% (3)	7.1% (1)

*The answers are presented aggregated per section in the questionnaire*.

a *Number of the response countries that have the respective control program in place*.

b *Percentage of countries that have chosen the answers “Data exist,” “No existing data,” “Unknown” out of the number of respondents in each section of the online data collection tool [number of countries/number of responding countries (n)]*.

Data relating to IBR and BVD CPs were reported as existing in more than 65% of the countries with CPs for these diseases (12 and 11 countries, respectively), and existence of data related to JD CPs was much lower (36%) (Table 1).

**Figure 3:**
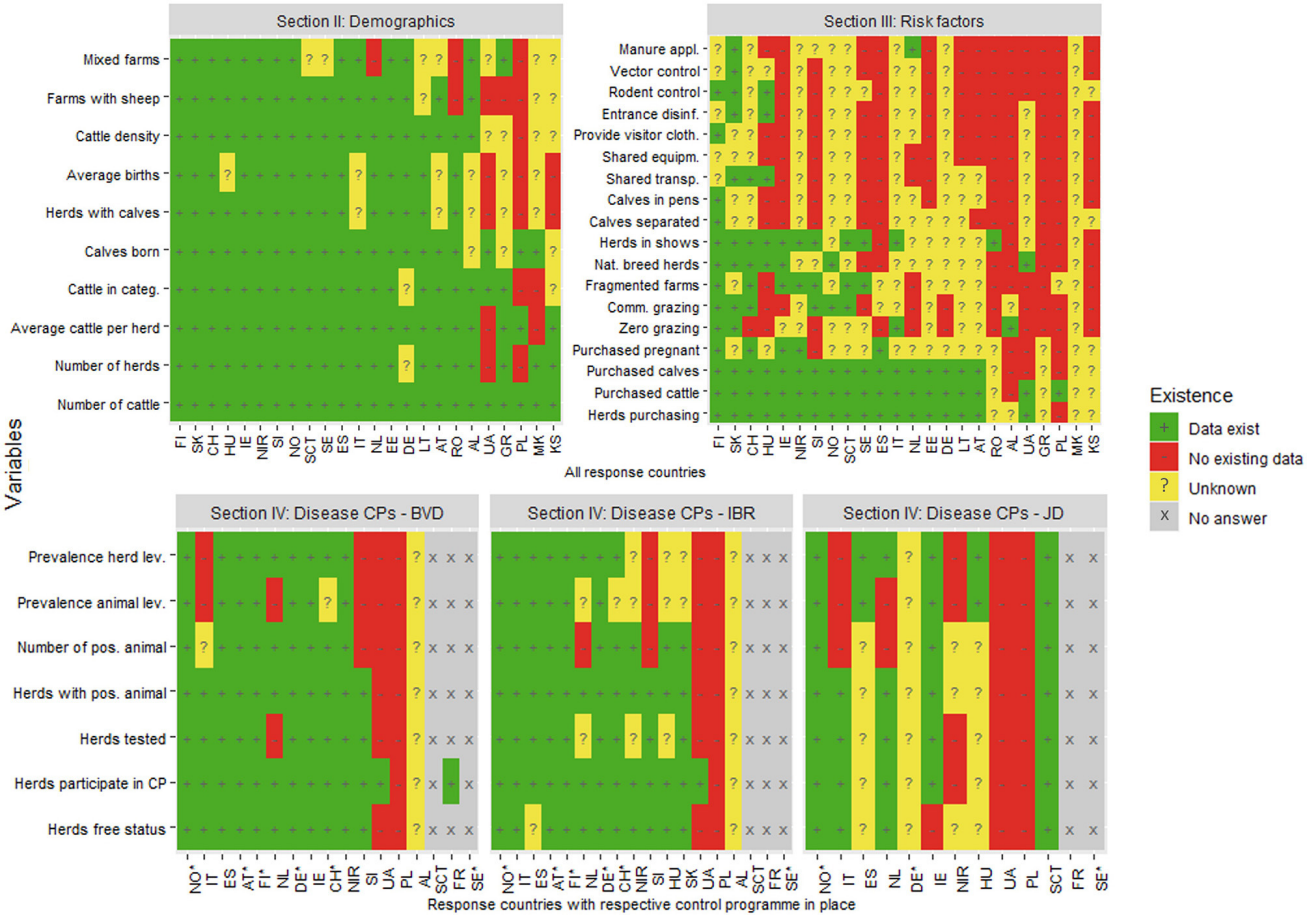
The existence of data relevant for estimating probability of freedom from infection in cattle in an online questionnaire study among 24 European countries. The answers are presented by country and variable. ^*^free from disease status. FI, Finland; SK, Slovakia; CH, Switzerland; HU, Hungary; IE, Ireland; NIR, Northern Ireland (UK); SI, Slovenia; NO, Norway; SCT, Scotland (UK); SE, Sweden; ES, Spain; IT, Italy; NL, Netherlands; EE, Estonia; DE, Germany; LT, Lithuania; AT, Austria; RO, Romania; AL, Albania; UA, Ukraine; GR, Greece; PL, Poland; MK, North Macedonia; KS, Kosovo.

The types of data existing in each responding country are presented in Figure 3. All responding countries reported having data about the number of cattle although only 30% of the countries had information about mixed farms. In the section of risk factors, around 75% of the countries reported purchase data as existing, and the rest of the data were “unknown” and “not existing.” Almost all the data about CPs of BVD and IBR were reported as existing in around 75% of the countries with the implemented CPs for these diseases. For JD, on the other hand, data about the CP existed in 50% of those countries with a CP

### The Quality of Existing Data

The results about the overall quality of existing data in SOUND control countries are presented in Table 2. More than 60% of the existing demographics and disease control program data was evaluated by the respondents as “good,” and a small proportion of these data were evaluated as “fair” or “poor.” The overall data quality was lower for data related to risk factors: only 33% were assessed as “good,” and 50 and 17% of risk factors data evaluated as “fair” and “poor,” respectively.

**Table 2:** Results of the response to questions on overall quality of data relevant for estimating freedom from infection in cattle in an online questionnaire study among 24 European countries.

**Section**			**Overall quality**		
			**Good**^**b**^	**Fair**^**b**^	**Poor**^**b**^
II. Demographics		(*n =* 18)	77.8% (14)	16.7% (3)	5.5% (1)
III. Risk factors		(*n =* 6)	33.3% (2)	50.0% (3)	16.7% (1)
IV. Disease control programs^a^	JD	(*n =* 5)	60.0% (3)	20.0% (1)	20.0% (1)
	IBR	(*n =* 12)	58.3% (7)	25.0% (3)	16.7% (2)
	BVD	(*n =* 11)	72.7% (8)	18.2% (2)	9.1% (1)

*The answers are presented aggregated per section in the questionnaire*.

a *Number of the response countries that have the respective control program in place*.

b *Percentage of countries that had existing data and chose the answer “Good,” “Fair,” “Poor” for each variable in the online data collection tool out of the number of respondents in each section of the online data collection tool [number of countries/number of responding countries (n)]*.

**Figure 4:**
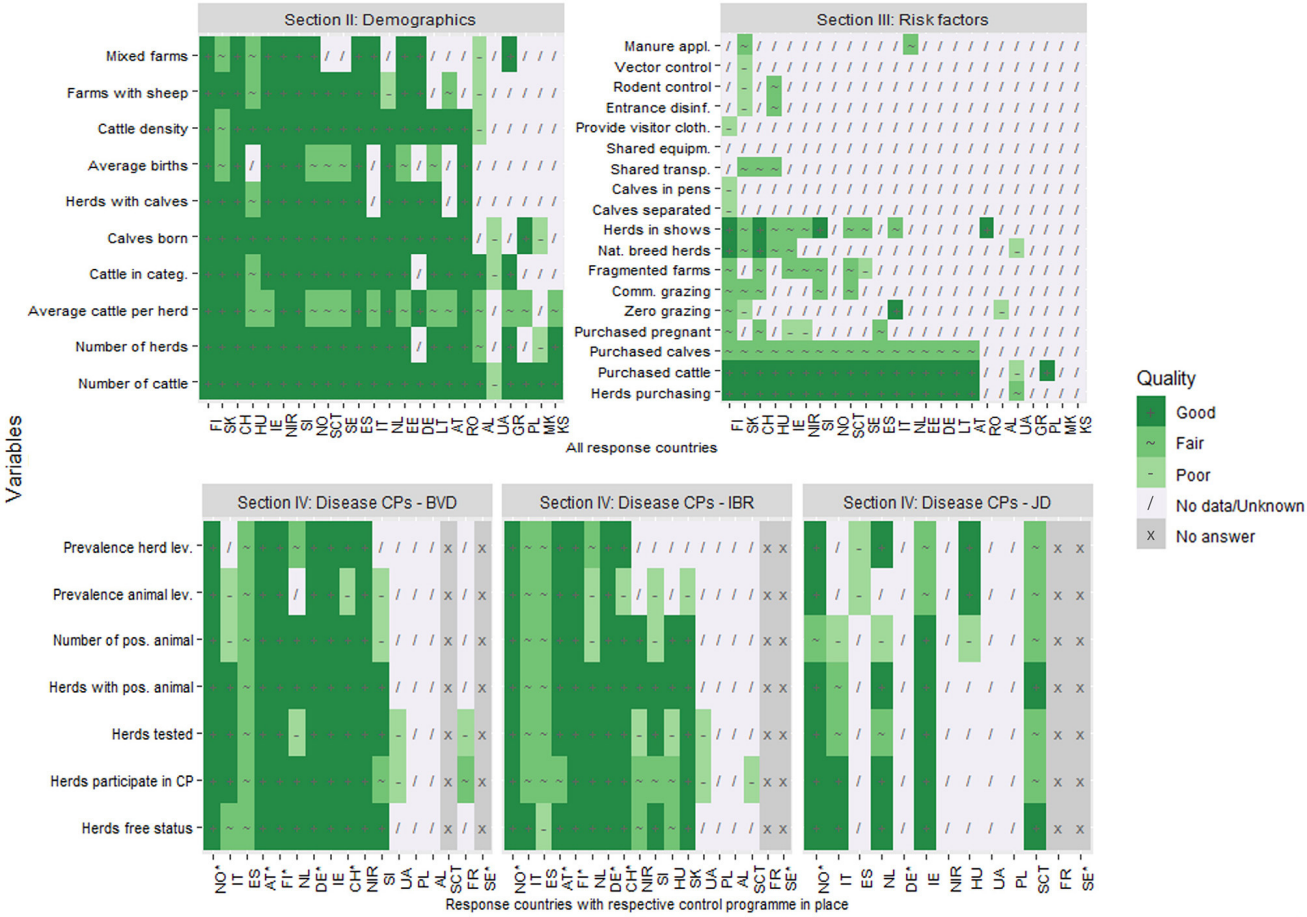
The overall quality of data relevant for estimating probability of freedom from infection in cattle in an online questionnaire study among 24 European countries. The answers are presented by country and variable. ^*^free from disease status. FI, Finland; SK, Slovakia; CH, Switzerland; HU, Hungary; IE, Ireland; NIR, Northern Ireland (UK); SI, Slovenia; NO, Norway; SCT, Scotland (UK); SE, Sweden; ES, Spain; IT, Italy; NL, Netherlands; EE, Estonia; DE, Germany; LT, Lithuania; AT, Austria; RO, Romania; AL, Albania; UA, Ukraine; GR, Greece; PL, Poland; MK, North Macedonia; KS, Kosovo.

The results were quite consistent within each country in terms of data quality in all four sections of the data collection tool (Figure 4). Around three quarters of demographics data in all response countries were evaluated as “good.” However, the quality of the average number of births and cattle per herd rated lower in more than half of the countries. A reverse picture of the quality score can be seen in the risk factors section as only data describing cattle purchase was consistently evaluated as “good” in all countries but one. Data on other existing risk factors was given lower quality scores.

Data quality for all evaluated criteria are presented in Table 3. An average percentage of “good” quality data in each section is consistent by criterion. The lowest quality score for data from all sections was the score for completeness. On the other hand, timeliness data were given the highest quality score. Additionally, among the disease CPs, the quality of BVD data was evaluated highest and JD lowest within all four quality criteria.

**Table 3:** Results of the response to questions on quality of data by criterion relevant for estimating freedom from infection in cattle in an online questionnaire study among 24 European countries.

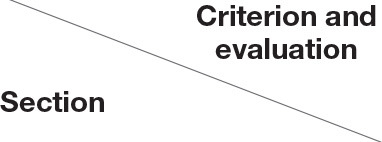			**Accessibility**			**Timeliness**		
			**Good**^**b**^	**Fair**^**b**^	**Poor**^**b**^	**Good**^**b**^	**Fair**^**b**^	**Poor**^**b**^
II. Demographics		(*n =* 18)	55.6% (10)	33.3% (6)	11.1% (2)	55.6% (10)	38.9% (7)	5.5% (1)
III. Risk factors		(*n =* 6)	16.7% (1)	50.0% (3)	33.3% (2)	50.0% (3)	16.7% (1)	33.3% (2)
IV. Disease control programs^a^	JD	(*n =* 4)	50.0% (2)	25.0% (1)	25.0% (1)	50.0% (2)	25.0% (1)	25.0% (1)
	IBR^c^	(*n =* 10)	77.8% (7)	11.1% (1)	11.1% (1)	80.0% (8)	10.0% (1)	10.0% (1)
	BVD	(*n =* 10)	80.0% (8)	10.0% (1)	10.0% (1)	80.0% (8)	10.0% (1)	10.0% (1)
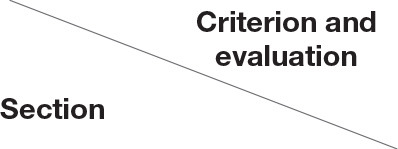			**Accessibility**			**Timeliness**		
			**Good**^**b**^	**Fair**^**b**^	**Poor**^**b**^	**Good**^**b**^	**Fair**^**b**^	**Poor**^**b**^
II. Demographics		(*n =* 18)	61.1% (11)	27.8% (5)	11.1% (2)	50.0% (9)	44.4% (8)	5.6% (1)
III. Risk factors		(*n =* 6)	16.7% (1)	50.0% (3)	33.3% (2)	33.3% (2)	50.0% (3)	16.7% (1)
IV. Disease control programs^a^	JD	(*n =* 4)	50.0% (2)	25.0% (1)	25.0% (1)	50.0% (2)	25.0% (1)	25.0% (1)
	IBR	(*n =* 10)	70.0% (7)	20.0% (2)	10.0% (1)	50.0% (5)	50.0% (5)	0.0% (0)
	BVD	(*n =* 10)	70.0% (7)	20.0% (2)	10.0% (1)	60.0% (6)	30.0% (3)	10.0% (1)

*The answers are presented aggregated per section in the questionnaire*.

a *Number of the response countries that have the respective control program in place*.

b *Percentage of countries that had existing data and chose the answer “Good,” “Fair,” “Poor” by criteria and each variable in the online data collection tool out of the number of respondents in each section of the online data collection tool [number of countries/number of responding countries (n)]*.

c *For accessibility n = 9*.

### Variety of Sources Used to Obtain the Same Data

**Figure 5:**
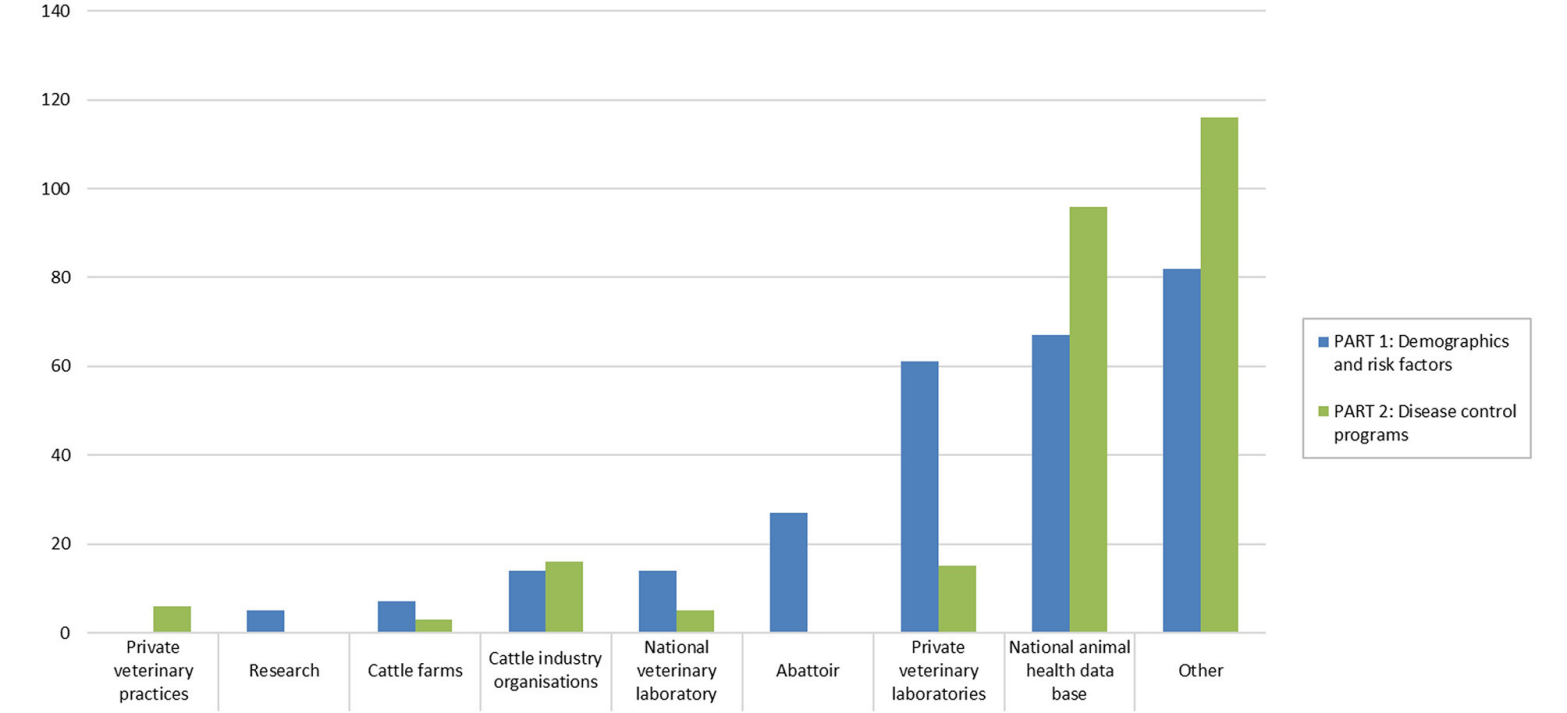
Sources used to obtain data relevant for estimating probability of freedom from infection in cattle in an online questionnaire study among 24 European countries. The answers are presented aggregated per part in the questionnaire.

The respondents used a variety of sources to assess and obtain the requested data. On average, two different sources of data were selected to answer one question. The most selected data source option in all sections was “other” followed by “national animal health databases” (Figure 5). The sources of data most frequently reported under the category “other” were agriculture statistics, central statistics databases, livestock registration databases, national animal traceability databases, and CPs databases.

## Discussion

Our study provides the first overview of data availability and quality related to estimating freedom from infection in 24 European countries. In addition, an overview of data availability and quality regarding control programs implemented for three major EU non-regulated cattle diseases, i.e., BVD, JD, and IBR was provided. Previous similar studies that aimed to describe the cattle sector or disease CPs in Europe have included fewer countries (6, 30). As participation in this study was voluntary, not all invited countries fully completed the questionnaire; however, the response rate was high (75%, 24 out of 32 invited countries). Nevertheless, the response rate was likely influenced by the COVID-19 pandemic and could even have been higher in another situation because many veterinary epidemiologists or veterinary public health specialists involved in the project were also actively participating in the emergency response to the pandemic.

### The Availability of Data for Probability of Freedom From Disease Estimates

A high proportion of the requested data about cattle demographics (75%) was reported as existing in the response countries. This section included general statistical information about the cattle sector, i.e., average herd size, number of calves born, etc. As having a computerized database for tracing individual bovines is a requirement within the EU (EC 1760/200), it is expected that this data is routinely collected within the response countries (31). Based on the responses, it appears that sufficient information about general demographics is available to describe the cattle industry within the responding countries and to use these data as parameters for modeling freedom from disease (e.g., average herd size, number of animals). In contrast, only around one fourth (24%) of the requested data about risk factors were reported to exist. The existing data on risk factors were mainly related to cattle purchase, which was homogenously reported as existing in response countries. This is most likely because these data are mandatory to report under guidelines of the European Commission TRACES system (32). Data about less regulated risk factors for disease introduction, such as herds involved in communal grazing, shared transport, etc., were, on the other hand, mostly non-existent or unknown. Risk factors for introduction of disease play an important role in estimating the probability of freedom (16). To facilitate future development of output-based standards for CPs, more data related to this topic need to be collected on a large scale in a systematic manner and made available for scientific use. Nevertheless, animal movement data (i.e., number of animals imported), and prevalence of infection in the farm of origin might be sufficient to obtain a first estimate of freedom from infection as seen, for example, in a study defining output-based standards for tuberculosis in farmed deer (11). Given the amount of data available, future models developed to compare outputs of various CPs could focus on the usage of nearly any data describing cattle demographics and cattle purchase data for risk of introduction of disease (16). However, although the introduction of cattle into a herd has been reported as an important risk factor for JD, IBR, and BVD in several studies (33–35), other risk factors, such as participation in shows, grazing, and calving pen systems, are also described as important for estimating freedom from infection (33). Our results show that very few countries in Europe have access to these data on a national or regional level.

Many countries have implemented CPs for the three diseases considered in our analysis. Most data in those countries were reported as existing for BVD (72%) and IBR (66%) and less for JD (33.8%). Six of the countries with CPs claimed having freedom from IBR, four from BVD, and two for JD (23). Countries that reported having CPs in place and/or being free from the disease also reported noticeably more data existing than countries with no CPs and/or no free status in sections on demographics and risk factors. Knowing that demonstrating freedom from disease requires sufficient scientific evidence that the disease is truly absent in the country, we could assume that those countries have developed efficient systems collecting more disease-specific data that is required to monitor and establish those statuses (36, 37).

### The Quality of Data Available for Probability of Freedom From Disease Estimates

Our results show that 50% of existing data were evaluated as “good” for all four quality criteria except for data related to risk factors (Table 3). In general, if the input data for freedom from disease estimations are good quality, results of those estimations could also be considered as good and accurate.

The criterion “timeliness” was evaluated as “poor” or “fair” by almost 40% of the responding countries meaning that a lot of the available data were not updated on a regular basis and do not reflect the current situation of the country, which may be an issue when estimating the probability of freedom from infection. Similarly, about 45% of the data were assessed as “fair” or “poor” in terms of accessibility, meaning that the data were not freely available for use. In addition, almost 50% of the data were evaluated as having poor or fair accuracy. Such lower evaluation indicates that data may be collected without any or only some validation procedures applied, leading to less credible information. Finally, more than half of the data had poor or fair completeness. This result could indicate that the data we aimed to assess likely come from sources where such information is not mandatory to report, leading to incomplete data sets and some missing values.

Ideally, all four data quality criteria evaluated in our study should be good quality to get as accurate as possible freedom from infection estimates. However, as mentioned in our results, completeness and accuracy were generally evaluated lower compared with timeliness and accessibility. Low data quality in terms of completeness and accuracy is significant as using data that comes from incomplete data sets and/or data that does not reflect the true values could lead to inaccurate estimates. Poor accessibility and timeliness, on the other hand, do not impact the ability to accurately estimate freedom of disease. However, they pose practical difficulties as data is not easy to obtain and does not reflect the most current or “real-time” probability of freedom from disease.

The overview about the existence and quality could be used as an indicator of which data are easily accessible and which are not. In addition, existing data evaluated as “poor” or “fair” should be interpreted with care when modeling freedom from infection, and care should be taken to explore the uncertainty associated with the outputs of the model. If the quality of data is considered especially important in output-based surveillance, and high-quality data are unavailable, actions should be taken to address this gap. However, assessing the requirements was not part of this study. Still, this study does provide an indication of the gaps and possibilities for improvement when aiming to collect comparable data from many European countries.

### Future Perspectives for the Online Data Collection Tool and Collected Data

Our online data collection tool was initially converted from Excel spreadsheets to an online questionnaire using survey-making software “Limesurvey.” However, it is difficult to assess how sustainable this form of data collection is. One option to consider would be to move toward an individual database-like tool, to which countries would be able to submit data and receive the outcome. Another prospect could be to optimize the data-collection process and collect required data from primary sources directly to the database or model, similar to the results of the SIGMA project (21). In addition, data that were reported as not existing in most of the countries, i.e., more than 50%, could be excluded from the data collection tool as such data would not support a wide implementation of output-based standards in Europe. To fill this gap, further work of SOUND control will focus on identifying the data that is necessary for output-based surveillance, and a joint research agenda for future research will be developed. Future work should also consider availability and validity of diagnostic tools as well as inherent differences in transmission dynamics among the diseases in question. The questions on diagnostic strategies in this study could potentially provide a basis for this, but a higher response rate would be required to evaluate them. Finally, in this data collection, respondents were able to voluntarily submit quantitative data that was not analyzed in this study (Figure 1C). Quantitative data that were submitted by respondents will further assist in developing and testing the first models for probability of freedom from disease in an output-based framework.

### Limitations of the Study

The current study has limitations in the assessment of the existence and quality of data that could lead to possible bias. First, when a respondent reports “no existing data,” the information may exist although it was unknown to the respondent. Similarly, “unknown” data may actually reflect non-existing data. However, if data exist and were easily accessible, the respondent would have most likely found it. As such, both “unknown” and “non-existing” data may represent not easily accessible or not well-described data. Nevertheless, this data collection was conducted within the framework of SOUND control, and our targeted respondent in each country are experts in the field of animal health surveillance, which increases the likelihood of the respondent being aware of existing data sources. Second, uncertainties in data quality evaluation could lead to false final outputs of the models (i.e., probability of freedom from infection), e.g., when data was mistakenly evaluated as good. This could also be an issue when discussing whether the probability of freedom from infection reflects the true situation of the country. Although respondents provide definitions of each quality criteria when assessing them, it is possible that they were interpreted differently by different respondents. Observed difficulties in data quality evaluation were related to the requested data being available from several sources, meaning that one source with the data of interest was easily accessible, however containing less accurate data compared with another, less accessible source with more accurate data. In cases such as this, it was unclear which data source to select and provide scores in the data quality evaluation tool. Thus, data evaluations (as good/fair/poor) should not be used as absolute values and be interpreted with care, and future studies further validating consistency of answers to the criteria would be useful. Finally, the results of the current study mainly represent country-level data on the cattle industry, risk factors for disease introduction, and CPs relevant to non-regulated cattle diseases. Therefore, respondents indicated that some data is collected on a regional level, but there is no centralized database from which to take the relevant data. In future studies, this data collection framework could be used to collect the data related to non-regulated cattle diseases in order to have a summarized overview of data originating from multiple sources.

## Conclusion

With data from 24 EU countries, our work provides an overview of the current situation in Europe in terms of data related to the EU non-regulated cattle diseases. This study further identified gaps in data availability, such as risk factor occurrence, which indicates where further work is needed. A standardized system of output-based surveillance could offer valuable evidence of animal health status to countries engaging in the trade of live cattle. For this approach to be optimized, it is necessary that those countries that would benefit from the information that such a system can provide should take steps to collate and share relevant data so that the estimates are as accurate as possible and the system achieves its potential. Overall, this work provides input for the next a step toward an output-based framework.

## Data Availability Statement

The raw data supporting the conclusions of this article will be made available by the authors, without undue reservation.

## Ethics Statement

This study was conducted within the COST Action (CA 17110), Standardizing OUtput-based surveillance to control Non-regulated Diseases in the EU (SOUND control) that was approved by the EU Committee of Senior Officials through written procedure on 13 April 2018 [Memorandum of Understanding (MoU) (22)]. The members of SOUND control that were involved in the current study formally accept the Action's MoU and did not require the study to be reviewed or approved by an ethics committee. In addition, the members of SOUND control who provided the data used for the study are co-authors of this paper. They have all reviewed and approved the content of the manuscript.

## Author Contributions

ER prepared the manuscript, which was revised primarily by CFa, L-MT, GS, and AR. Conceptual contributions were made by IS-B, XK, and MM. Provision of data for the study and editing the final version of the manuscript were made by JG, BC, TK, JH, JB, LPC, AMad, AT, AG, AMal, BS, CFo, EK, F-FR, HH, KD, KM, LÓ, LC, MG-G, MH, MA, NP, PH, RJ, SS, RM, SV, and TA. All authors contributed to the article and approved the submitted version.

## Conflict of Interest

CFa was employed by the company Ausvet Europe. The remaining authors declare that the research was conducted in the absence of any commercial or financial relationships that could be construed as a potential conflict of interest.
